# Unusual Thermal Transport in Few‐Layer Van der Waals Antiferromagnet CrOCl

**DOI:** 10.1002/advs.202502440

**Published:** 2025-04-25

**Authors:** Yu Yang, Yan Zhou, Ziming Tang, Yulu Liu, Weimin Quan, Jun Zhou, Xiaokang Li, Xiaoxiang Xi, Qihua Gong, Lifa Zhang, Yunshan Zhao

**Affiliations:** ^1^ Phonon Engineering Research Center of Jiangsu Province Ministry of Education Key Laboratory of NSLSCS Center for Quantum Transport and Thermal Energy Science Institute of Physics Frontiers and Interdisciplinary Sciences School of Physics and Technology Nanjing Normal University Nanjing 210023 China; ^2^ College of Physics and State Key Lab of Mechanics and Control for Aerospace Structures and Key Lab for Intelligent Nano Materials and Devices of Ministry of Education and College of Aerospace Engineering Nanjing University of Aeronautics and Astronautics (NUAA) Nanjing 210016 China; ^3^ National Laboratory of Solid State Microstructures and Department of Physics Nanjing University Nanjing 210093 China; ^4^ Wuhan National High Magnetic Field Center and School of Physics Huazhong University of Science and Technology Wuhan 430074 China

**Keywords:** magnetic thermal transport, magnon‐phonon coupling, antiferromagnetic, interfacial thermal transport

## Abstract

Few‐layer van der Waals magnets are exceptional candidates for investigating the fundamental spin behaviors and advancing the development of next‐generation ultra‐compact spintronic devices. While the intrinsic long‐range magnetic order is well‐established in the monolayer limit, the thermal transport behavior involving magnons, phonons, and magnetophonon polarons near the phase transition remains largely unexplored. In this work, the thermal transport behavior is probed near the phase transitions from bulk to the monolayer limit by using a differential suspended thermal bridge method, which provides an ultra‐sensitive temperature and thermal conductance measurement enhanced by the double Wheatstone bridge. In the few‐layer CrOCl flake, a stronger magnon‐phonon coupling is observed compared to the bulk, resulting in a shift in the thermal transport behavior from a dip to a peak shape around the Néel temperature. Additionally, below the Néel temperature, the few‐layer CrOCl significantly enhances the interfacial thermal conductance between the metal electrode and insulator substrate, potentially leading to the substantial improvements in the heat dissipation in Si‐based semiconductor devices. This study introduces a novel method and strategy for probing the fundamental magnetic phase transition behavior and lays a solid foundation for the potential application of van der Waals magnets in the electronic devices.

## Graphical abstract

Thermal transport serves as an ultra‐sensitive probe for detecting the magnetic order in 2D materials. Using the suspended thermal bridge method enhanced by Wheatstone bridges, it is, for the first time, observed anomalous critical behavior in few‐layer CrOCl near its Néel temperature. Meanwhile, the first experimental confirmation is provided that magnon can enhance interfacial thermal conduction in metal‐insulator interface.

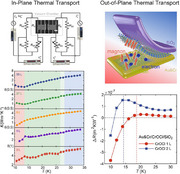

## 1 Introduction

Spintronics, which enables the active control and manipulation of the spin degree of freedom, is expected to drive the next generation of electronic devices, offering smaller, faster, and more energy‐efficient data storage and processing technologies.^[^ ^1^ ^]^ Compared to the ferromagnetic (FM) materials, the antiferromagnetic (AFM) materials are widely regarded as a promising candidate for future spintronics devices due to their robustness against external magnetic field disturbances, no stray fields, ultrafast dynamic response, and large magnetotransport effect.^[^ ^2^ ^]^ However, the complex interaction between spin, charge, and lattice vibration within AFM materials presents challenges in identifying the magnetic candidates with optimal performance.^[^ ^3^ ^]^ To overcome this obstacle, the thermal transport measurements have been employed as a sensitive probe to study the elementary spin excitations in novel magnetic materials, as they are particularly effective at reflecting the relaxation times and scattering strengths of the low‐energy excitations. Consequently, a wide range of spin excitations have been investigated, including FM spin excitation in Yttrium Iron Garnet,^[^ ^4^, ^5^ ^]^ AFM spin excitation in cuprates^[^ ^6^, ^7^ ^]^ and manganates,^[^ ^8^ ^]^ Kitaev spin liquid states in RuCl_3_^[^ ^9^, ^10^ ^]^ and Na_2_Co_2_TeO_6_,^[^ ^11^, ^12^ ^]^ phonon‐glass‐like behavior in Tb_2_Ti_2_O_7_,^[^ ^13^ ^]^ metallic spin ice state in Pr_2_Ir_2_O_7_,^[^ ^14^ ^]^ and unconventional spin excitations in multiferroic materials like YMnO_3_^[^ ^15^ ^]^ and Fe_2_Mo_3_O_8_,^[^ ^16^ ^]^ etc. Due to the complexity of spin ground states, most of these bulk AFM systems exhibit a series of highly sensitive magnetic‐field‐dependent and temperature‐dependent phases, making it challenging to achieve stable and high‐performance spintronics devices.

According to the Mermin–Wagner theorem, the spontaneous magnetization does not exist in the 2D isotropic Heisenberg model at finite temperatures.^[^ ^17^ ^]^ However, the intrinsic low lattice symmetry of 2D van der Waals (vdW) layered magnetic materials introduces the natural magnetic anisotropy, opening a gap in the low‐energy excited magnon modes. This enhances the potential for discovering long‐range magnetic order in 2D systems, driven by various spin exchange interactions between atoms or electrons, such as direct and indirect exchanges, asymmetric Dzyaloshinsky–Moriya (DM) interactions, and Ruderman–Kittel–Kasuya–Yosida (RKKY) interactions, etc.^[^ ^18^, ^19^, ^20^, ^21^ ^]^ Furthermore, spin‐orbit coupling further enhances the magnetic anisotropy by coupling the spin and lattice vibrations, making the magnetic contribution to the thermal conductivity closely tied to the lattice contribution—a phenomenon that has garnered significant attention in recent years. In the vdW magnets, whether FM or AFM materials, the thermal conductivity is strongly suppressed by the spin‐phonon scattering around the magnetic ordering temperature *T*_c_ or *T*_N_.^[^ ^9^, ^10^, ^22^, ^23^, ^24^, ^25^, ^26^, ^27^, ^28^, ^29^, ^30^, ^31^ ^]^ Typically, a slightly dip or plateau‐type region is observed at or below the magnetic ordering temperature due to the large population of thermally excited magnons, which leads to a strong magnon‐phonon scattering, often overshadowing the magnon contributions. As the temperature decreases further, the population of magnons significantly diminishes due to the anisotropic gap at the zone center of magnetic excitations, causing the thermal conductivity to gradually revert to a phonon‐dominated trend. Interestingly, the suppression of thermal conductivity persists even when the temperature exceeds or far exceeds the magnetic ordering temperature. In this temperature region, the long‐range magnetic order is destroyed and the short‐range spin fluctuations begin to form, continuing to suppress the thermal conductivity through the spin‐phonon interaction. As the temperature rises, the coherence, lifetime, and interaction distance of magnons weaken, gradually reducing this supression, leading to a finite increase in thermal conductivity, and eventually transitioning to a normal phonon transport behavior governed by Umklapp scattering.^[^ ^8^, ^13^, ^14^, ^15^, ^25^, ^30^, ^31^, ^32^ ^]^ During this process, the thermal conductivity often exhibits abnormal fluctuation behavior, commonly referred to as the glasslike thermal transport region^[^ ^25^, ^33^, ^34^, ^35^, ^36^ ^]^ in previous studies. This spin‐suppressed thermal conductivity can be alleviated through the application of an external magnetic field, potentially increasing the total thermal conductivity severalfold.^[^ ^22^, ^23^, ^24^, ^25^ ^]^

In 2017, the existence of long‐range magnetic order was confirmed in monolayer (ML) and few‐layer (FL) samples of 2D vdW materials CrI_3_^[^ ^37^ ^]^ and Cr_2_Ge_2_Te_6_,^[^ ^38^ ^]^ which opened up new opportunities for the development of novel 2D spintronics devices. As the thickness of 2D vdW materials is reduced to the monolayer limit, their thermal transport properties undergo significant changes.^[^ ^39^ ^]^ For instance, the thermal conductivity of ML graphene can reach up to 3000 Wm^−1^ K^−1^, nearly ten times that of its bulk counterpart.^[^ ^40^, ^41^ ^]^ Naturally, this raises questions about the thermal transport properties of 2D vdW magnetic materials in FL and ML forms, and how magnetism influences their thermal transport. While the intrinsic long‐range magnetic order has been well‐established at the monolayer level, the thermal transport behavior involving magnons, phonons, and magnetophonon polarons near the phase transition remains largely unexplored.

The vdW AFM insulator CrOCl, a representative of transition metal oxygen halides, has received extensive attention due to its air stability, ease of mechanical exfoliation, and the variety of physical phenomena it exhibits.^[^ ^42^, ^43^, ^44^, ^45^, ^46^, ^47^, ^48^, ^49^, ^50^, ^51^, ^52^, ^53^, ^54^, ^55^, ^56^, ^57^, ^58^, ^59^, ^60^, ^61^, ^62^, ^63^, ^64^, ^65^, ^66^, ^67^ ^]^ The unique low‐symmetry staggered square lattice of CrOCl imparts an inherent magnetic frustration, leading to a stable quasi‐1D stripy magnetic order at low temperatures. Hence, CrOCl exhibits a rich array of magnetic phases,^[^ ^42^, ^43^, ^44^, ^45^, ^46^, ^47^, ^48^, ^49^, ^50^ ^]^ including antiferromagnetic state, incommensurate state, paramagnetic state, and a wide range of physical effects, such as strong spin‐phonon coupling, spin density wave, spin Peierls effect, and the magnetic proximity effect.^[^ ^50^, ^51^, ^52^, ^53^, ^54^, ^55^, ^56^ ^]^ Additionally, various spectroscopic phenomena^[^ ^57^, ^58^ ^]^ and applications in photodetection^[^ ^59^, ^60^, ^61^ ^]^ have been observed in CrOCl. However, the stable AFM ground state found in the bulk CrOCl remains debated in the ML, with different DFT calculations suggesting either FM^[^ ^62^, ^63^ ^]^ or AFM^[^ ^64^, ^65^ ^]^ ground states. Furthermore, there are discrepancies between the theoretical calculation^[^ ^66^ ^]^ and the experiment measurement^[^ ^67^, ^68^ ^]^ regarding the room temperature thermal conductivity, and currently, no experimental data is available on a few layers CrOCl at low temperatures to support the theoretical debate on its magnetic ground state.

In this work, we report the unconventional spin‐lattice coupled thermal conductivity of atomically thin CrOCl with the thickness from bilayer (BL) to the bulk limit. In the FL samples, a distinct peak is observed at the Néel temperature *T*_N_, which starkly contrasts with the temperature‐dependent thermal conductivity observed in the bulk CrOCl and other magnetic materials. Additionally, we have observed, for the first time, an anomalous enhancement in the interfacial thermal transport between the FL CrOCl and Cr/Au electrodes as the temperature drops below the Néel temperature *T*_N_, which may serve as a direct evidence that the magnon can enhance the interfacial thermal transport. Our findings provide important insights into the thermal conductivity of the atomically‐thin vdW AFM CrOCl as well as the interfacial magnon‐carrier interaction.

## 2 Results and Discussion

### 2.1 Material Characterizations

**Figure 1:**
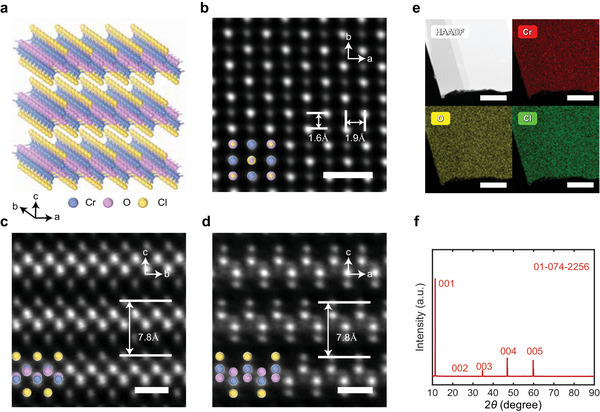
Structural characterization of vdW layered CrOCl single crystals. a) Schematics of the crystallographic structure of vdW layered CrOCl single crystals. b) Atomic‐resolution HADDF STEM image of a CrOCl single crystal, viewed along the [001] direction. The scale bar is 0.5 nm. c,d) Atomic‐resolution cross‐sectional HADDF STEM images of CrOCl single crystal, viewed along the [100] and [010] directions. The scale bar is 0.5 nm. e) The HAADF image of the analysed area with corresponding elemental mapping for Cr, O and Cl. The scale bar is 200 nm. f) The XRD pattern of CrOCl single crystal.

CrOCl is a vdW AFM insulator with a staggered square lattice and a band gap of 2.3 eV.^[^ ^47^, ^48^, ^49^, ^50^ ^]^ As illustrated in **Figure** **1**a, CrOCl belongs to the *D*_2h_ point group, and has a crystal structure composed of Cr–O double layers sandwiched between two Cl atoms along ***c***‐axis at room temperature. Two nonequivalent Cr atoms each form a distorted octahedron with four neighboring O atoms and two neighboring Cl atoms. These nonequivalent Cr atoms form two different staggered‐arrangement Cr–Cl–Cr chains along the ***b***‐axis, introducing the spin frustration to the lattice. Owing to the stability of the CrOCl crystals, a spherical aberration‐corrected scanning transmission electron microscope (AC‐STEM) is employed to quantitatively reveal the atomic arrangement of a mechanically exfoliated FL CrOCl flake. A series of the high‐angle annular dark‐field (HAADF) and bright‐field (BF) STEM images at different magnifications clearly reveal the atomic ordering of CrOCl in the ***ab*** plane, as shown in Figure 1b and Figure S1 (Supporting Information). A brighter contrast of Cr columns over O/Cl columns can be distinctly identified in HAADF mode, which matches well with the previously known atom arrangement. Furthermore, due to the intrinsic lattice anisotropy, the crystal orientation of the ***a***‐axis and ***b***‐axis can be determined by measuring two lattice parameters perpendicular to each other, yielding values of *a* = 3.2 Å and *b* = 3.8 Å at room temperature. The selected‐area electron diffraction (SAED) pattern of a larger sample region, shown in the subfigure of Figure S1c (Supporting Information), is consistent with our X‐ray diffraction (XRD) result (Figure 1f). The five distinct XRD peaks indicate a preferred growth orientation of CrOCl crystals along the ***c***‐axis ([001] axis). Additionally, a large area of energy dispersive X‐ray spectroscopy (EDS), shown in Figure 1e, demonstrates a uniform distribution of the Cr, O, and Cl elements, reflecting the high quality and homogeneity of our flake. The cross‐sectional HAADF STEM images along ***a*** and ***b*** axes, shown in Figure 1c,d, and Figures S2 and S3 (Supporting Information), further reveal the atomic‐resolution layer structure of CrOCl. These images clearly display the staggered chains of Cr atoms, allowing for accurate measurement of interlayer lattice parameters, where *c* = 7.8 Å.

Additionally, we conducted the spectroscopic characterization of CrOCl flakes. An optical image of the sample used for Raman spectroscopy measurement is shown in Figure S4 (Supporting Information), and the corresponding contour color map of angularly resolved polarized Raman spectroscopy (ARPRS) is shown in Figure S5 (Supporting Information). The Raman spectrum reveals three strong peaks at 208.5 cm^−1^ ($A_{g}^{1}$), 415.2 cm^−1^ ($A_{g}^{2}$), 457.1 cm^−1^ ($A_{g}^{3}$) at room temperature.^[^ ^49^, ^50^ ^]^ These three A_g_ modes exhibit a periodicity of 180° under the parallel polarization and 90° under the perpendicular polarization, as clearly demonstrated in Figure S6 (Supporting Information). Moreover, X‐ray photoelectron spectroscopy (XPS) is employed to determine the bonding states of the CrOCl crystal. As shown in Figure S7 (Supporting Information), the XPS peaks of Cr 2*p* spectrum appear at 577.15, 578.35, and 587.65 eV, corresponding to Cr^3 +^ 2*p*_3/2_, Cr^3 +^ 2*p*_3/2_, and Cr^3 +^ 2*p*_1/2_, respectively. The O 1*s* spectrum shows a peak at 532.95 eV, while the Cl 2*p* spectrum presents peaks at 199.65 eV (Cl 2*p*_3/2_) and 201.25 eV (Cl 2*p*_1/2_).

### 2.2 Phase Transition Behavior of CrOCl at Low Temperature

Similar to TiOCl,^[^ ^69^, ^70^ ^]^ the presence of two closely spaced Cr stripy chains in CrOCl facilitates the direct exchange interactions, leading to an unusual phase diagram and a series of complex phase transitions. As the temperature decreases, the paramagnetic CrOCl undergoes two pure‐magnetically‐induced second‐order phase transitions at ≈27 K, potentially forming an incommensurate spin density wave (SDW) state along the ***b***‐axis. Upon reaching the Néel temperature *T*_N_ of 13.5 K, CrOCl undergoes a first‐order phase transition, accompanied by a twofold lattice and structural distortion, resulting in a uniaxial antiferromagnetic state with an easy axis along the ***c***‐axis.^[^ ^48^, ^50^ ^]^ This transition is driven by the strong magnetoelastic coupling, which induces a spin‐Peierls transition, causing CrOCl to shift from the orthorhombic space group *Pmmn* to the monoclinic space group *P2_1_/m*. As a result, two Cr stripy chains are formed, accompanied by a 2 ***b*** nuclear superstructure and an intralayer fourfold magnetic superstructure with an unique monoclinic symmetry along the ***a***‐axis.^[^ ^44^, ^45^, ^46^, ^47^, ^48^ ^]^ These complex phase transitions have been confirmed through various experimental methods, including the heat capacity measurement,^[^ ^47^, ^50^ ^]^ the magnetic moment analysis,^[^ ^42^, ^43^, ^45^, ^50^ ^]^ XRD,^[^ ^46^, ^47^, ^49^ ^]^ Raman spectroscopy,^[^ ^49^, ^50^ ^]^ tunneling magnetic conductance (TMC),^[^ ^45^, ^48^, ^50^ ^]^ relative permittivity studies^[^ ^45^ ^]^ and the dynamic cantilever magnetometry.^[^ ^44^ ^]^

Before measuring the thermal transport properties of CrOCl, we employed a combination of experimental methods to explore its physical behavior around the phase transitions. Temperature‐dependent tunneling magnetoconductance devices (see Experimental Section for details) are used to determine the phase transition temperatures in FL CrOCl, as they are particularly sensitive to the magnetic phase transitions in FL magnets.^[^ ^48^, ^50^ ^]^ Two FL CrOCl samples are connected to cross‐structured FL graphene strips, as shown in Figures S8 and S9 (Supporting Information). As the longitudinal voltage increases, the insulated CrOCl gradually becomes conductive, and the tunneling magnetoconductance rapidly increases, which can be described by Fowler–Nordheim tunneling model^[^ ^48^, ^50^ ^]^:

(1) $$\ln\;\frac{I}{V^{2}}=\alpha \frac{\phi_{B}^{3/2}}{V}$$

**Figure 2:**
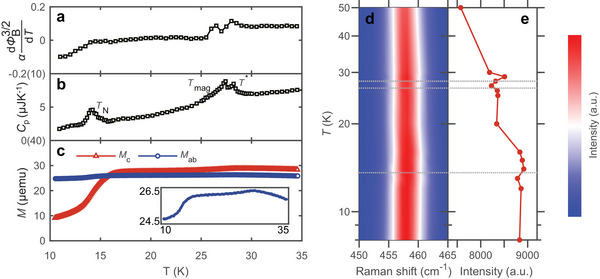
The phase transition characterization of CrOCl. a–c) Temperature‐dependent tunneling magnetoconductance, heat capacity and magnetic moment of CrOCl. d) Temperature‐dependent Raman spectroscopy of $A_{g}^{3}$ mode in parallel polarized configuration. e) The strength of $A_{g}^{3}$ mode under different temperatures.

Here, α represents a constant, *I* and *V* represent the tunneling voltage and tunneling current, and ϕ_*B*_ represents the tunneling barrier height. The original measurement data are presented in Figure S9 (Supporting Information). Using Equation (1), the data in the high tunneling voltage region are linearly fitted to obtain the temperature‐dependent $\phi_{B}^{3/2}$, as shown in Figure S10 (Supporting Information). At the three‐phase transition temperatures, $\phi_{B}^{3/2}$ exhibits a discontinuous trend. To precisely identify the phase transition temperatures, we plot the temperature derivative of $\alpha \phi_{B}^{3/2}$, which is $\alpha \frac{d\phi_{B}^{3/2}}{dT}$, in **Figures** **2**a and S10 (Supporting Information). At both *T* ^*^ and *T*_mag_, $\alpha \frac{d\phi_{B}^{3/2}}{dT}$ shows two distinct peaks, while at *T*_N_, $\;\alpha \frac{d\phi_{B}^{3/2}}{dT}$ begins to decrease significantly. Although the noise is stronger in thin tunneling magnetoconductance devices due to the small breakdown voltage and significant thermal expansion effect, both tunneling magnetoconductance devices yield the phase transition temperatures that are consistent with the heat capacity *C*_p_ of the bulk CrOCl, as shown in Figure 2b. Additionally, the temperature‐dependent magnetic moment of bulk CrOCl with fields parallel and perpendicular to the ***c***‐axis is also characterized, similar to the results reported previously^[^ ^42^, ^43^, ^47^, ^49^ ^]^ as shown in Figure 2c and Figure S11 (Supporting Information). As the temperature decreases, a broad peak appears ≈28 K, and the magnetic moments begin to decrease in both directions, which corresponds to the magnetically induced phase transitions and short‐range magnetic order occurring at *T* ^*^ and *T*_mag_. Above *T*_N_, ≈17 K, the magnetic moments in both directions decrease significantly. Then, the ***c***‐axis magnetic moment dropping by ≈60% at *T*_N_, indicating a long‐range AFM order with an easy axis close to the c‐axis.

Additionally, the temperature‐dependent Raman spectroscopy with different polarization configurations is employed to study the phase transition behavior of CrOCl, as shown in Figure 2d,e, Figures S12 and S13 (Supporting Information). A noticeable change in both the peak intensity of $A_{g}^{3}$ mode under parallel polarization is observed. Two distinct intensity saturations and blue shifts of $A_{g}^{3}$ mode at ≈27 and 17 K indicate the occurrence of the phase transitions and the presence of spin‐phonon coupling.^[^ ^49^, ^50^ ^]^ Simultaneously, we observed a significant electronic contribution in the low wave number region, causing the Raman baseline to increase as the temperature decreases.^[^ ^71^, ^72^ ^]^ Consequently, we also collected the temperature‐dependent Raman spectra under different polarization configurations, revealing a spectral weight transfer from low frequency to high frequencies, which may be associated with the opening of an SDW gap in low temperature.

### 2.3 In Plane Thermal Transport Measurement

**Figure 3:**
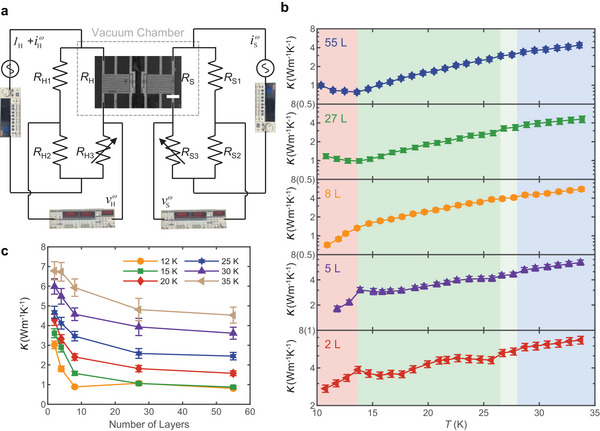
The in plane thermal transport of CrOCl. a) The electrical circuit of the double Wheatstone bridge enhanced suspended thermal bridge method. The scale bar is 10 µm. b) Temperature‐dependent in‐plane thermal conductivity of CrOCl. c) Layer‐dependent in‐plane thermal conductivity of CrOCl under different temperatures.

Here, to accurately measure the thermal transport behavior near the phase transition temperature, we develop a high‐sensitivity, low‐temperature‐bias measurement scheme for the suspended 2D materials. This method, which utilizes a double‐Wheatstone bridge‐enhanced suspended thermal bridge with high temperature sensitivity (see Method S1, **Figure** **3**a, Figures S15 and S16 for details, Supporting Information), was employed to measure the thermal conductivity of CrOCl at low temperatures. The device and its measurement results are shown in Figure 3b,c, Figures S14–S17 (Supporting Information). Three unusual behaviors of thermal conductivity are observed at all phase transition temperatures. Around the Néel temperature *T*_N_ = 13.6 K, a strong magnetoelastic coupling and lattice distortion induce a spin‐Peierls transition, resulting in a significant suppression of thermal conductivity in the thicker samples, similar to the previous findings in other bulk AFM materials.^[^ ^13^, ^22^, ^23^, ^24^, ^27^, ^29^, ^32^ ^]^ Below the *T*_N_, we observe only a slight increase in the thermal conductivity with no further decrease. However, in the 2L and 5L CrOCl samples, a distinct peak appears at *T*_N_, which is markedly different from the behavior in bulk samples. And this peak diminishes with increasing thickness and completely disappears in 8L sample. Interestingly, while the overall trend for the 8L sample is similar to that for the FL sample, no dip or peak is observed near the *T*_N_. Instead, a blunt bulge connects the different temperature‐dependent thermal conductivity between the AFM state and unconventional spin state. Thus, as the number of layers increase, we observe that the thermal conductivity behavior evolves from a clear peak to a blunt bulge, and eventually to a broad dip near *T*_N_. Since this work represents the first measurement of the thermal conductivity in a suspended FL 2D magnetic material, there are no direct experimental comparisons available. We believe this behavior may be attributed to the following factors: First, due to the symmetry selection rule, thin 2D materials exhibit a smaller scattering phase space and weaker scattering of flexural phonon modes, which can lead to an increased thermal conductivity as thickness decreases, as seen in some 2D materials.^[^ ^39^ ^]^ This effect is further amplified in suspended devices, allowing more ZA phonons to participate in thermal transport, thereby influencing the strong magnetoelastic coupling in CrOCl. In Figure 3c and Figure S18 (Supporting Information), we plot the thermal conductivity of all the samples, revealing a clear thickness effect, with thinner samples exhibiting significantly higher thermal conductivity. A second possible reason is the layer‐dependent magnetism. While bulk CrOCl is known to exhibit AFM order at low temperatures, the magnetic order in ML CrOCl remains debated. Some calculations suggest FM order,^[^ ^62^, ^63^ ^]^ while others propose it remains AFM.^[^ ^64^, ^65^ ^]^ Compared to the AFM order, the FM order may have distinct effects on the thermal transport, resulting in different magnetoelastic coupling phenomena. However, there are currently no comprehensive theoretical or experimental studies on how the magnetic order in CrOCl changes with decreasing layers, making it challenging to fully understand the thermal transport in thinner CrOCl.

In addition to the Néel temperature *T*_N_, we also observe two subsequent phase transitions near 27 K at *T* ^*^ = 28 K and *T*_mag_ = 26.5 K. These transitions result in slight discontinuities in the thermal conductivity around their respective phase transition temperatures, although the overall magnitude of the conductivity does not change significantly. In the 2L sample, a relatively clear suppression of the thermal conductivity, similar to that observed near *T*_N_ in thicker samples, is detected around *T*_mag_, suggesting that these two phase transitions are closely related to the initial establishment of the magnetic order. Meanwhile, within the temperature range between *T*_mag_ and *T*_N_, the thermal conductivity of FL CrOCl exhibits a glass‐like phenomenon, as previously reported in other studies.^[^ ^25^ ^]^ One possible cause of this glass‐like thermal transport is the short‐range spin‐phonon interaction caused by the low‐energy spin fluctuations.^[^ ^15^ ^]^ The two most significant suppressions occur at 17 and 27 K, indicating that the thin sample demonstrates a significant short‐range magnetic order at 27 K and a more pronounced longer‐range magnetic order at 17 K. This just coincided with the notable change in the magnetic moment near 17 K, indicating that the magnon‐phonon coupling in the FL magnets is stronger than that in the bulk magnets, where even minor changes in magnetic order are more readily reflected in the thermal conductivity. This observation provides a clearer understanding of the peak‐shaped characteristics of magnetic materials that appear near critical temperatures. In fewer‐layer CrOCl, we anticipate an even stronger magnon‐phonon interaction (MPI), which manifests in a shift of the central temperature of MPI (denoted as *T*_MPI_) to higher temperature and an increase in the interaction strength.^[^ ^25^ ^]^ However, when the temperature falls below *T*_MPI_, the population of thermal‐excited magnons with strong MPI decreases, while the contribution of the magnons to the thermal conductivity increases, leading to an abnormal enhancement of thermal conductivity near the Néel temperature *T*_N_. As the temperature decreases further, the population of carrier continues to decrease, leading to a subsequent reduction in the thermal conductivity. Overall, a peak‐shaped characteristic can be observed across the entire temperature range. For the thicker flake, *T*_MPI_ may approach the minimum temperature in our measurement system, leading to the observation of a dip‐shaped characteristic instead of a peak, consistent with the most reported bulk vdW magnetic materials. Thus, the peak‐shaped characteristic in bulk samples may shift to lower temperature (typically below *T*_N_)^[^ ^22^, ^23^ ^]^ and may weaken^[^ ^22^, ^23^, ^27^, ^32^ ^]^ or even evolve into a slight distortion,^[^ ^24^, ^26^, ^29^ ^]^ due to the significant reduction in the thermal conductivity caused by temperatures. In addition, the in‐plane thermal conductivity of CrOCl at high temperature is measured as well, which are discussed in detail in Note S1 (Supporting Information).

### 2.4 Magnon Enhanced Interfacial Thermal Transport

**Figure 4:**
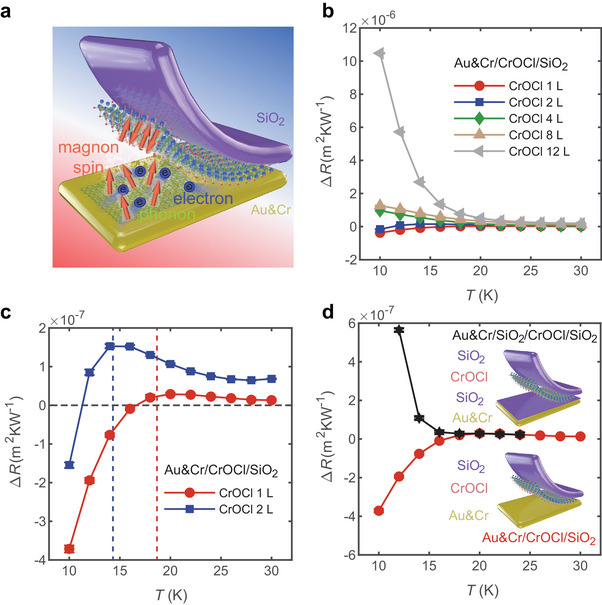
AFM magnon enhanced interfacial thermal transport. a) Schematics of interfacial thermal transport between ML CrOCl and metal electrode. b) The additional thermal resistance introduced by CrOCl flakes of varying the thicknesses between Au/Cr electrodes and SiO_2_/Si substrates. c) AFM magnon induced enhancement in the additional thermal resistance in the ML and BL CrOCl. d) The additional thermal resistance for Au&Cr/SiO_2_/CrOCl/SiO_2_ and Au&Cr/CrOCl/SiO_2_ configuration under different substrate temperatures. The oxidation layer suppresses the strong coupling between ML CrOCl and metal electrode.

The generation of magnetic order can significantly influence the in‐plane thermal transport in CrOCl, but what effect does it have on out‐of‐plane thermal transport? To investigate this, we measured the out‐of‐plane thermal conductivity of CrOCl using the differential 3ω method (see Experimental Section for details), as shown in **Figure** **4**a,b. The additional thermal resistance Δ*R* introduced by CrOCl flakes of varying thicknesses between Au/Cr electrodes and SiO_2_/Si substrates is measured and can be expressed as:

(2) $$\Delta R=R_{\mathrm{CrOCl}}^{\mathrm{intrinsic}}+R_{\mathrm{CrOCl}/{\mathrm{SiO}}_{2}}^{\mathrm{interface}}+R_{\mathrm{Cr}/\mathrm{CrOCl}}^{\mathrm{interface}}-R_{\mathrm{Cr}/{\mathrm{SiO}}_{2}}^{\mathrm{interface}}$$

For samples thicker than 3L, both the interfacial thermal resistance and intrinsic thermal resistance increase as the temperature decreases, leading to an increase in Δ*R*. This trend becomes more pronounced in the thicker sample. However, as shown in Figure 4c, in the ML and BL samples, the additional thermal resistance decreases unusually with decreasing temperature, and an unexpected enhancement in the interfacial thermal transport is observed below a certain temperature. For the ML sample, a sharp decrease in Δ*R* occurs at ≈17 K, corresponding to the initial temperature of the long‐range magnetic order in CrOCl. In the BL sample, a weak increase in the Δ*R* is observed before the unusual decrease, indicating that both the intrinsic thermal resistance and the interface thermal resistance are still present, while they are overshadowed by the stronger magnetic enhancement at lower temperatures. This magnetic enhancement is not observed in the thicker samples, possibly due to the proportion of intrinsic out‐of‐plane thermal resistance within the system gradually increases as the thickness further increases, which may mask or delay this effect.

In the ML devices, the intrinsic out‐of‐plane thermal resistance is very low, with most of the thermal resistance concentrated at the interface. To identify the source of the unusual enhancement, we introduce a SiO_2_ insulating layer (10 nm) between the Au/Cr electrode and the CrOCl flake to prevent the interaction between Cr and CrOCl, as shown in Figure 4d. When this insulating layer is added, the unusual enhancement disappears, indicating that the effect is related to the direct interaction between the Cr and CrOCl. This provides the first evidence of AFM magnon‐enhanced interfacial thermal conductance between a metal and AFM insulators, introducing a novel approach to enhancing interfacial thermal transport between metal and insulator. In contrast to the traditional metal interfacial materials and composite interfacial materials, CrOCl offers several unique advantages: high intrinsic thermal conductivity, high interfacial thermal conductance and insulation advantages. These attributes could play a crucial role in mitigating the thermal impact on integrated circuits, improving the heat dissipation capabilities of electronic device packages, and enhancing the stability of memory devices, light‐emitting diode (LED) devices, and high‐frequency communication devices, particularly at the interface between metal wires and Silicon‐On‐Insulator (SOI) structures within these devices.

At the metal‐AFM insulator interface, several carriers, including phonons, magnons, electrons, and possibly hybrid carriers, contribute to the heat transport, as shown in Figure 4a. As the temperature decreases, a long‐range AFM order gradually forms in CrOCl, participating the interfacial thermal transport. We hypothesize that the unusual magnetic enhancement may stem from the proximity‐effect‐induced interactions, such as the enhanced MPI or electron–phonon interactions at the interface. Given the current challenges in calculating interfacial thermal transport involving multiple types of carriers, it is difficult to directly simulate the thermal transport process in proximity magnetic systems. Instead, we employ the first‐principles calculations to explore the effect of the AFM order on the interfacial differential charge density distribution in a CrOCl/Cr interfacial system (see Methods for details). As shown in Figure S19 (Supporting Information), we discover a complex charge distribution at the CrOCl(AFM)/Cr interface, which does not increase the average differential charge density but results in a significant charge redistribution. This interfacial charge redistribution may be a key factor contributing to the unusual enhancement of interfacial thermal transport.

In conclusion, we have conducted the first study of the thermal transport in FL AFM CrOCl. Using a highly sensitive suspended thermal bridge method enhanced by double‐Wheatstone bridge, we are able to clearly observe the two adjacent magnetically induced phase transitions at *T* ^*^ and *T*_mag_. Meanwhile, we discover an unusual thermal conductivity behavior with an enhanced magnon‐phonon scattering in the BL CrOCl at *T*_N_. Furthermore, we report, for the first time, an AFM magnon‐induced enhancement of thermal transport at the metal/AFM insulator interface. Our research introduces new methods and opportunities for exploring FL vdW magnetic materials, and provides practical insights for addressing the thermal challenges in spintronic devices and Si‐based semiconductor devices.

## 3 Experimental Section

### Sample Characterizations

The high‐angle annular dark‐field (HAADF) scanning transmission electron microscopy (STEM) and bright‐field (BF) STEM images were obtained by a JEOL JEM‐ARM200F microscope equipped with a spherical aberration correction system for STEM, achieving the sub‐Å resolution. The energy‐dispersive X‐ray spectroscopy (EDS) mapping was performed with a 100 mm^2^ JEOL Centurio SDD EDS detector. Cross‐section HAADF STEM specimens were prepared using Zeiss Cross Beam 540 system. The room temperature angle‐resolved polarized Raman spectroscopy (ARPRS) measurements were conducted using a WiTec alpha 300R system. The ARPRS signal was collected through a Zeiss EC Epiplan‐Neofluar Dic 100× focus‐length objective with a high numerical aperture (0.9NA) and 600 grooves/mm grating (BLZ 500 nm). The laser power was maintained ≈10 mW and an excitation wavelength of 532 nm. The ARPRS measurement was conducted by rotating the sample while using a fixed half‐wave plate to achieve parallel or perpendicular polarization configurations. The temperature‐dependent Raman spectroscopy was measured using a Montana Instruments cryostat, with the samples mounted in a vacuum chamber during data acquisition. The laser power was kept at ≈7 mW with an excitation wavelength of 532 nm and each Raman spectrum was integrated over 10 s. Sample drift and laser spot defocusing were manually adjusted during measurements. The heat capacity was measured using a commercial measurement system (Quantum Design PPMS) while the magnetic moment measurements were conducted using a Quantum Design MPMS/SQUID. The thickness measurements were carried out using a Bruker Dimension Icon scanning probe microscope (atomic force microscope). The XPS was performed using the Thermo Fisher ESCALAB Xi+ system and the XRD measurements were conducted using the Rigaku‐SmartLab SE X‐ray Diffractometer with a μ‐XRD component.

### Fabrication and Measurement of Tunneling Magnetoconductance Devices and 3ω Devices

The CrOCl and graphene flakes of varying thicknesses were mechanically exfoliated onto a SiO_2_/Si substrate (285 nm oxide thickness) using the scotch tape under the ambient conditions. The uniform, rectangular CrOCl flakes were selected for fabricating 3ω devices after verifying their orientation through Raman spectroscopy. The tunneling magnetoconductance devices were assembled using a dry transfer technique, involving the stacking of polypropylene carbonate (PPC)/polydimethylsiloxane (PDMS) polymer stacks on a glass slide. Then, the standard electron‐beam lithography (EBL) was used to pattern the tunneling magnetoconductance and the 3ω electrodes. Electrodes were deposited by thermal evaporation of Cr/Au (5/65 nm), followed by a lift‐off process in acetone. The standard differential 3ω thermal transport and temperature‐dependent tunneling magnetoconductance measurements were performed in a cryostat system, with a minimum temperature of 10 K and a minimum pressure of 10^−7^ mbar. A LabVIEW program was used to automatically control the temperature, DC/AC sources and meters for the measurements.

### Density Functional Theory Calculations

The first‐principles calculations within the framework of density functional theory (DFT) were employed to analyse the electronic properties of CrOCl/Cr proximity system by using the Vienna Ab initio Simulation Package (VASP). The Perdew–Burke–Ernzerhof (PBE) generalized gradient approximation is used to solve electronic exchange‐correlation functional. To account for the strong‐correlated interaction, the Hubbard parameters *U* = 3 eV and *J* = 1 eV were applied to Cr atoms in CrOCl. During self‐consistent electronic optimization, a 5 × 5 × 1 Monkhorst–Pack *k*‐mesh was employed in the first Brillouin zone. A cutoff energy of 500 eV is set, and the convergence criterion for the total energy is set as 10^−5^ eV. A vacuum space of 30 Å along *z* direction was introduced to eliminate the interaction between adjacent slabs. A 4 × 2 CrOCl supercell was placed on two Cr (001) layers to study the potential impact of interfacial behavior on charge transfer. The lattice constants *a* and *b* of CrOCl were 3.26 and 3.95 Å, respectively, consistent with the experimental values. The charge density difference was calculated using the equation:

(3) $$\Delta \rho =\rho_{\mathrm{CrOCl}/\mathrm{Cr}-}\;\rho_{\mathrm{CrOCl}-}\rho_{\mathrm{Cr}}$$

where ρ_CrOCl/Cr_, ρ_CrOCl_ and ρ_Cr_ represent the charge densities of CrOCl/Cr proximity system, CrOCl, and Cr, respectively.

## Conflict of Interest

The authors declare no conflict of interest.

## Supporting information



Supporting Information

## Data Availability

The data that support the findings of this study are available from the corresponding author upon reasonable request.
