# Supplementary material for: Unusual Thermal Transport in Few‐Layer Van der Waals Antiferromagnet CrOCl
Source: Adv Sci (Weinh). 2025 Apr 25;12(26):2502440. doi: 10.1002/advs.202502440 (PMC12245046; doi:10.1002/advs.202502440)
Supplement: Supplementary file 1 — Supporting Information [file ADVS-12-2502440-s001.pdf]

ADVANCED SCIENCE
Open Access

Supporting Information

for *Adv. Sci.*, DOI 10.1002/advs.202502440

Unusual Thermal Transport in Few-Layer Van der Waals Antiferromagnet CrOCl

Yu Yang, Yan Zhou, Ziming Tang, Yulu Liu, Weimin Quan, Jun Zhou, Xiaokang Li, Xiaoxiang Xi, Qihua Gong, Lifa Zhang and Yunshan Zhao**

Supporting Information

Unusual thermal transport in few-layer van der Waals antiferromagnet CrOCl

Yu Yang[1], Yan Zhou[1], Ziming Tang[2], Yulu Liu[3], Weimin Quan[4], Jun Zhou[1], Xiaokang Li[4], Xiaoxiang Xi[3], Qihua Gong[2], Lifa Zhang[1,*] and Yunshan Zhao[1,*]

[1]Phonon Engineering Research Center of Jiangsu Province, Ministry of Education Key Laboratory of NSLSCS, Center for Quantum Transport and Thermal Energy Science, Institute of Physics Frontiers and Interdisciplinary Sciences, School of Physics and Technology, Nanjing Normal University, Nanjing 210023, China.

[2]College of Physics & State Key Lab of Mechanics and Control for Aerospace Structures & Key Lab for Intelligent Nano Materials and Devices of Ministry of Education & College of Aerospace Engineering, Nanjing University of Aeronautics and Astronautics (NUAA), Nanjing 210016, China.

[3]National Laboratory of Solid State Microstructures and Department of Physics, Nanjing University, Nanjing, 210093, China.

[4]Wuhan National High Magnetic Field Center and School of Physics, Huazhong University of Science and Technology, Wuhan, 430074, China

[*]Corresponding authors. E-mails: phyzys@njnu.edu.cn; phyzlf@njnu.edu.cn;

This PDF file includes:

Figure S1 to Figure S19

Method S1

Note S1

This PDF file includes:

Figure S1 Structural characterization of vdW layered CrOCl single crystals along [001] direction

Figure S2 Structural characterization of vdW layered CrOCl single crystals along [100] direction

Figure S3 Structural characterization of vdW layered CrOCl single crystals along [010] direction

Figure S4 Optical image of a CrOCl flake for Raman measurement on SiO2/Si substrate

Figure S5 The ARPRS characterization of CrOCl

Figure S6 The polar plots of the ARPRS characterization of CrOCl

Figure S7 The XPS spectra of CrOCl

Figure S8. The Measurement method schematic of temperature-dependent tunnelling magnetic conductance

Figure S9. Temperature- dependent tunnelling magnetic conductance of CrOCl

Figure S10. Phase transition associated tunneling magnetic conductance signal

Figure S11. The magnetic field dependence magnetic moment of bulk CrOCl

Figure S12. Temperature-dependent Raman spectroscopy of bulk CrOCl in different polarized configurations

Figure S13. Subtraction in Raman responses between various temperatures and 300K under different polarized configurations

Figure S14. Suspended thermal bridge devices for measuring thermal conductivity with different thickness CrOCl flakes

Figure S15. Temperature stability test of the double Wheatstone bridge enhanced suspended thermal bridge method

Figure S16. Measurement repeatability test of the double Wheatstone bridge enhanced suspended thermal bridge method

Figure S17. Finite element simulation of double Wheatstone bridge enhanced suspended thermal bridge method

Figure S18. The thermal conductivity of CrOCl with different thicknesses

Figure S19. Differential charge density distribution in CrOCl/Cr proximity systemMethod S1Untra-sensitive suspended thermal bridge enhanced by double Wheatstone bridge

Method S1 Untra-sensitive suspended thermal bridge enhanced by double Wheatstone bridge

Note S1 The in-plane thermal conductivity of CrOCl at high temperature

Figures

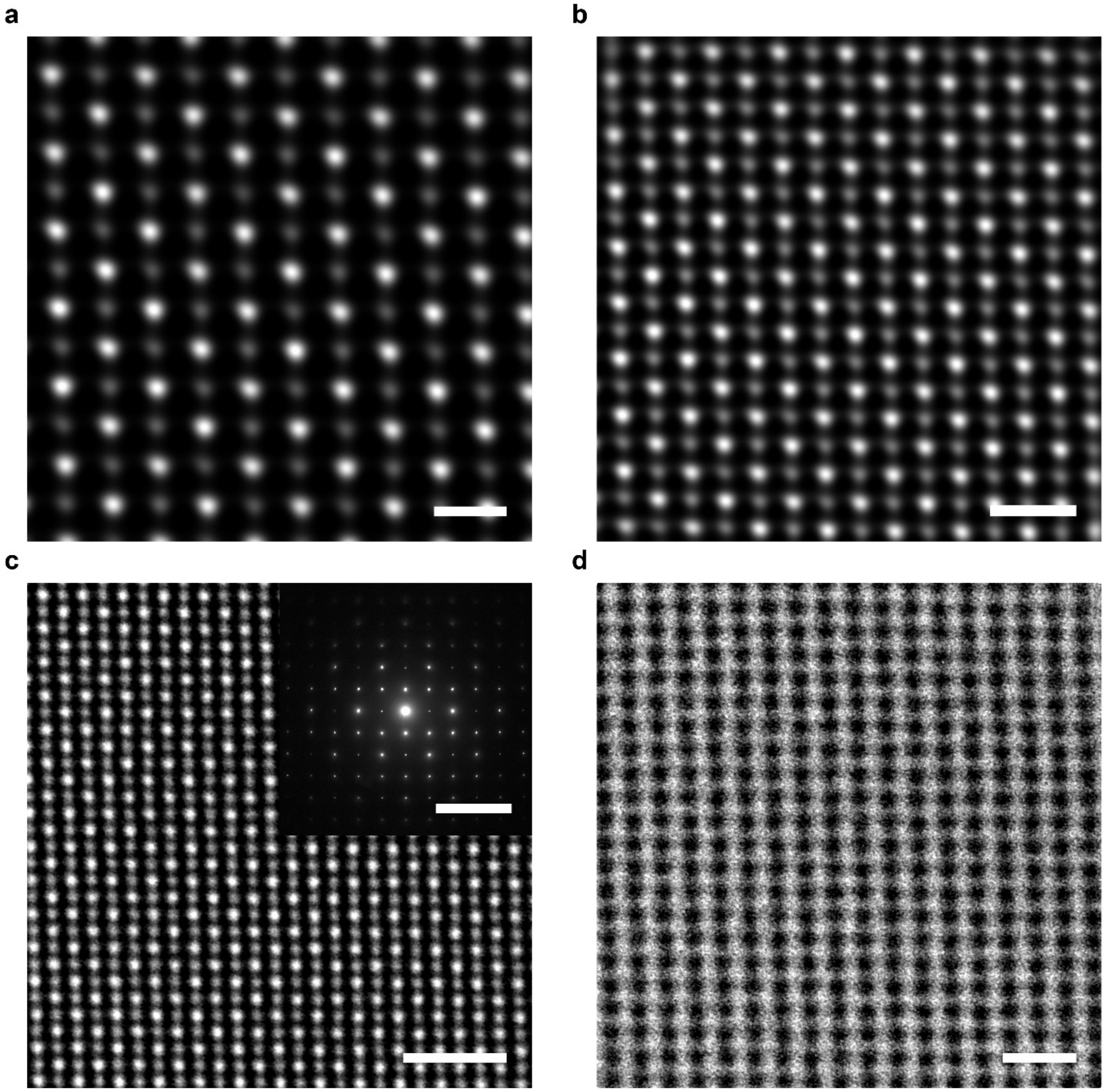

Figure S1. Structural characterization of vdW layered CrOCl single crystals along [001] direction. **a-c** Atomic-resolution HADDF STEM image of CrOCl single crystal, viewed from the [001] direction. The scale bar is 0.3 nm (**a**), 0.5 nm (**b**) and 1.0 nm (**c**), respectively. The SAED pattern of the Figure S1c is shown in its subfigure. The scale bar is 5 nm^{-1}. **d** Atomic-resolution BF STEM image of CrOCl single crystal, viewed from the [001] direction. The scale bar is 0.5 nm.

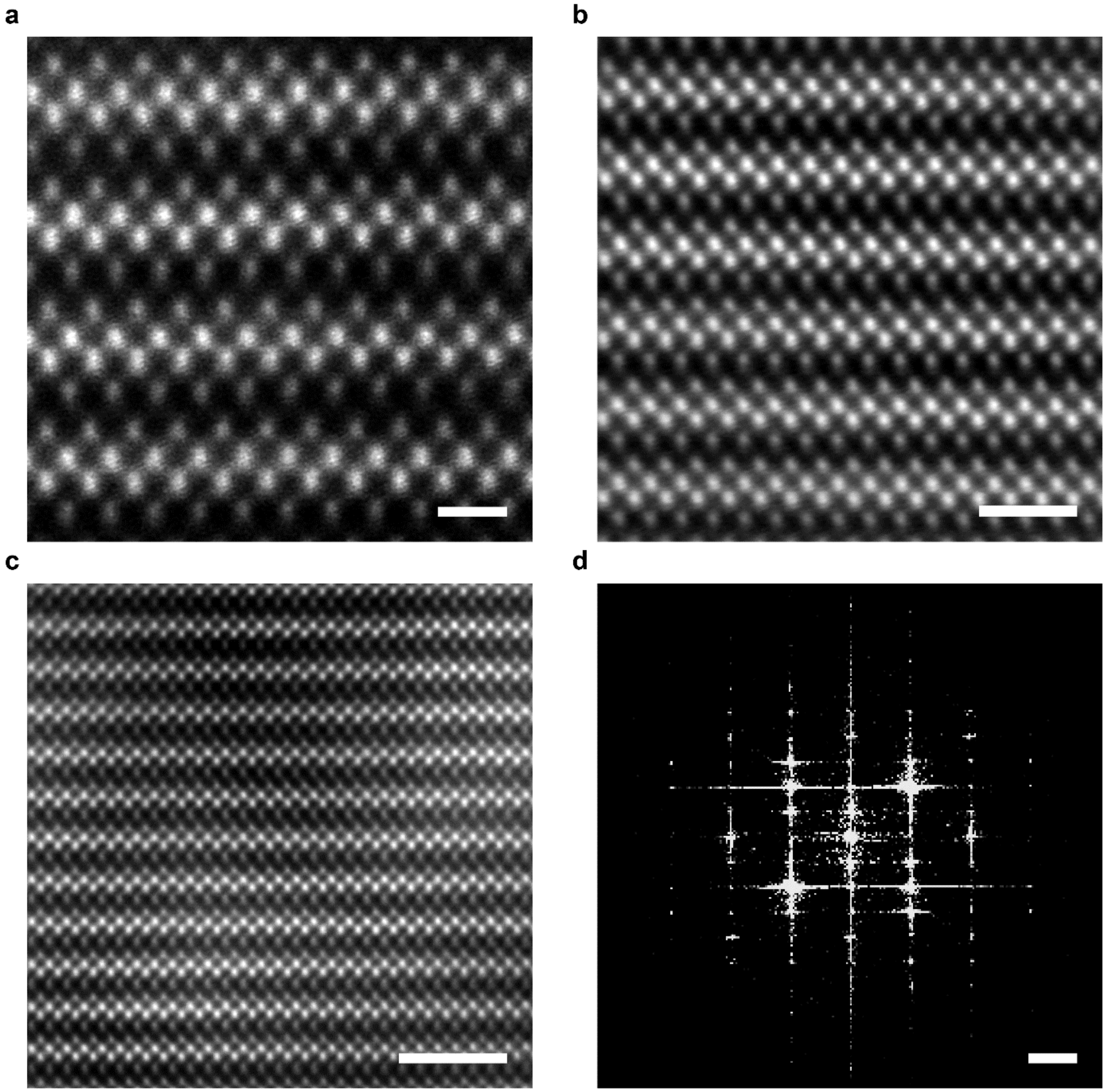

Figure S2. Structural characterization of vdW layered CrOCl single crystals along [100] direction. **a-c** Atomic-resolution HADDF STEM image of CrOCl single crystal, viewed from the [100] direction. The scale bar is 0.5 nm (**a**), 1.0 nm (**b**) and 2.0 nm (**c**), respectively. **d** The FFT pattern of the Figure S2c. The scale bar is 5 nm^{-1}.

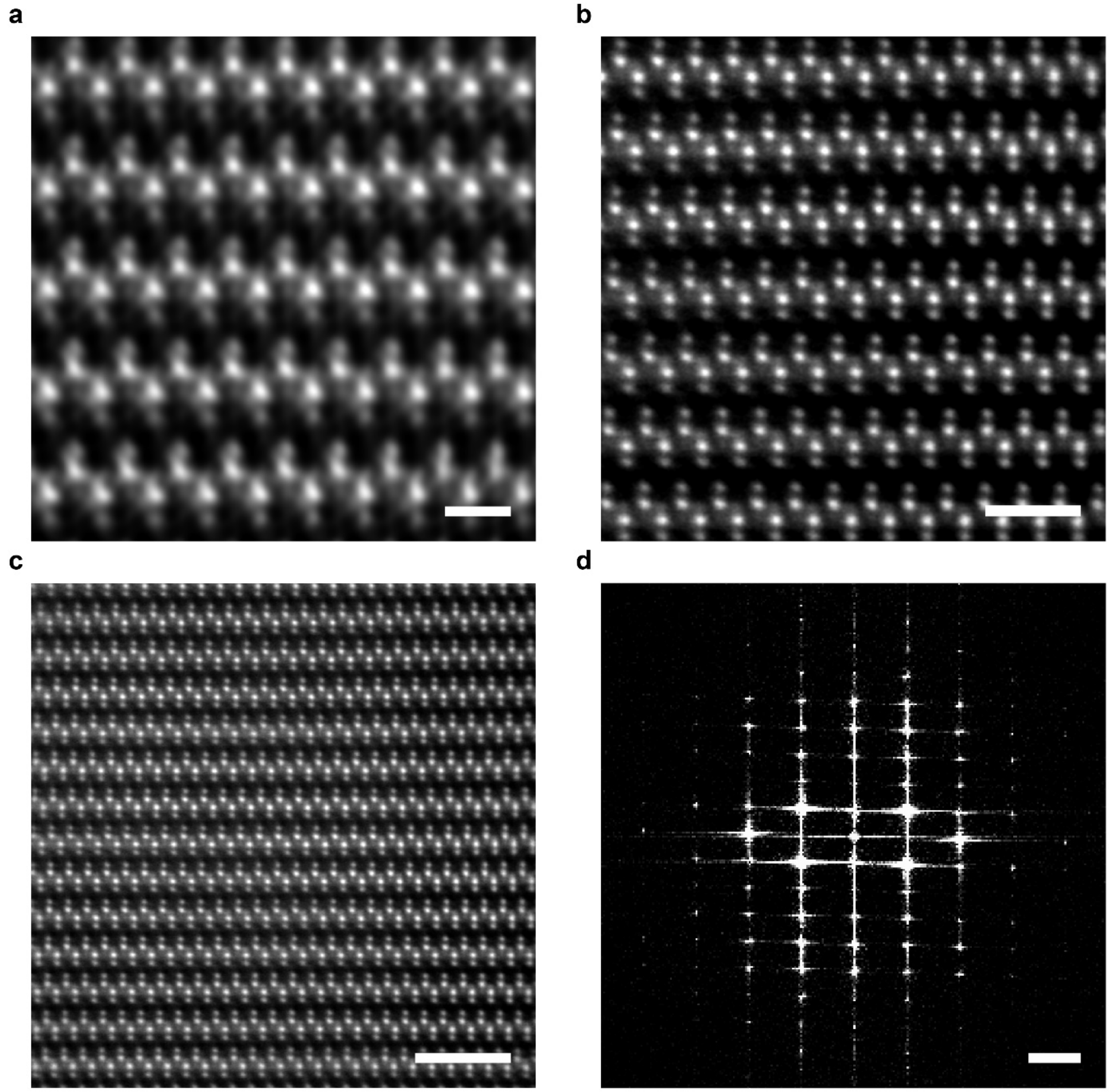

Figure S3. Structural characterization of vdW layered CrOCl single crystals along [010] direction. **a-c** Atomic-resolution HADDF STEM image of CrOCl single crystal, viewed from the [010] direction. The scale bar is 0.5 nm (**a**), 1.0 nm (**b**) and 2.0 nm (**c**), respectively. **d** The FFT pattern of the Figure S2c. The scale bar is 5 nm^{-1}.

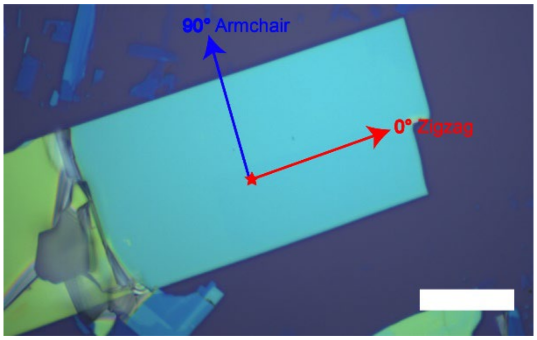

Figure S4. Optical image of a CrOCl flake for Raman measurement on SiO_2/Si substrate. The scale bar is 20 μm.

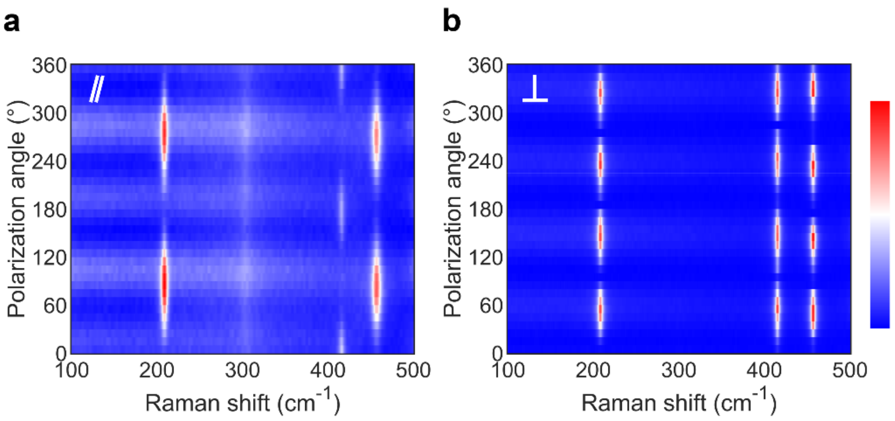

Figure S5. The ARPRS characterization of CrOCl. a-b Contour color map of normalized angle-resolved polarized Raman intensities under parallel (a) and perpendicular (b) polization configurations of CrOCl.

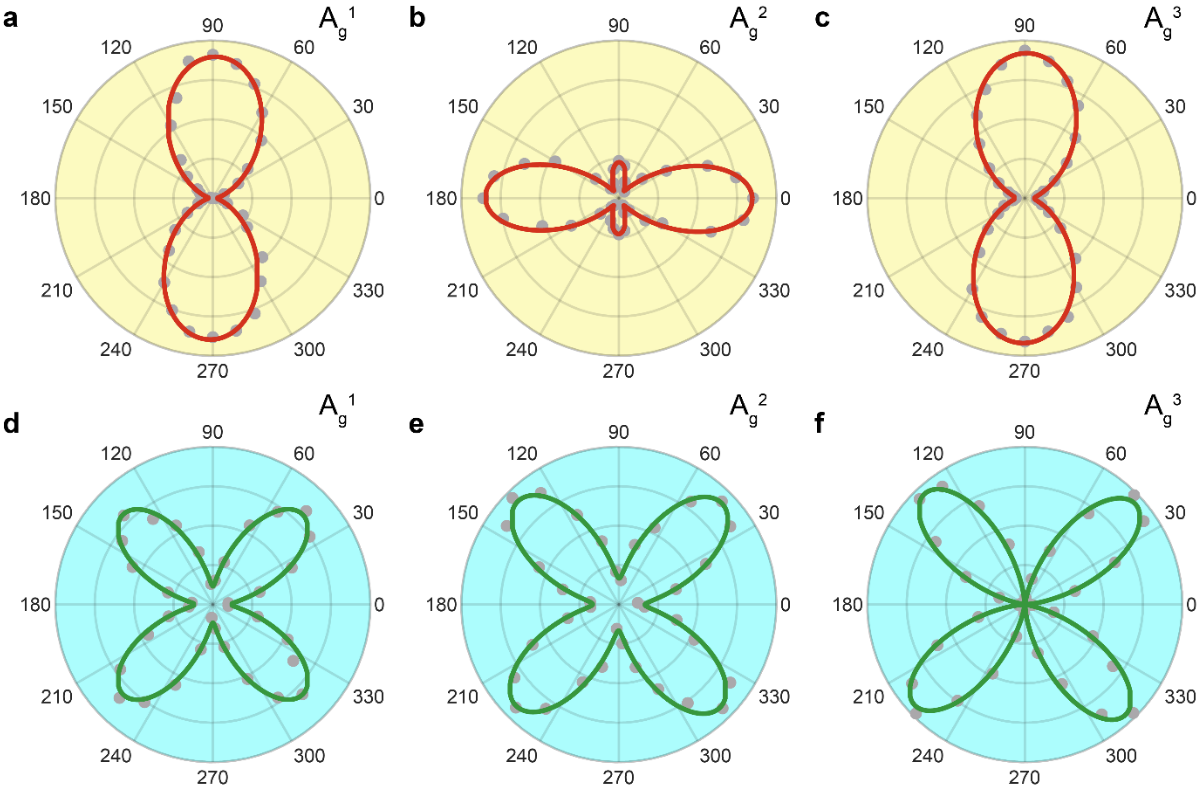

Figure S6. The polar plots of the ARPRS characterization of CrOCl. Polar plots of Raman intensity for A_g^1, A_g^2, and A_g^3 modes under parallel (a−c) and perpendicular (d−f) polization configurations. The gray dots and the solids lines are the experimental data and fitted curves.

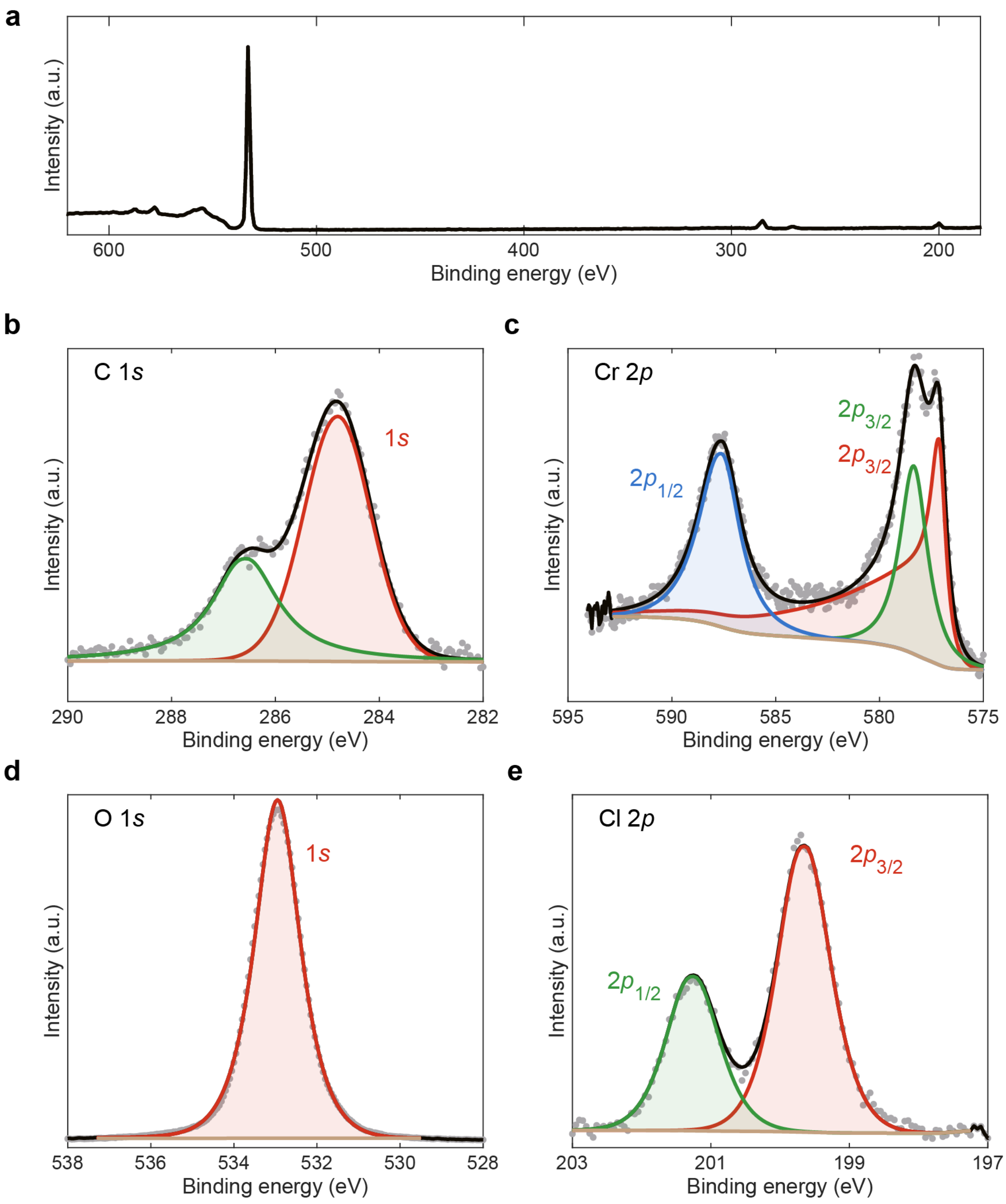

Figure S7. The XPS spectra of CrOCl. a The XPS full spectrum of CrOCl. **b-e** The XPS spectra of CrOCl in the 2 C 1*s*, Cr 2*p*, O 1*s* and Cl 2*p* region.

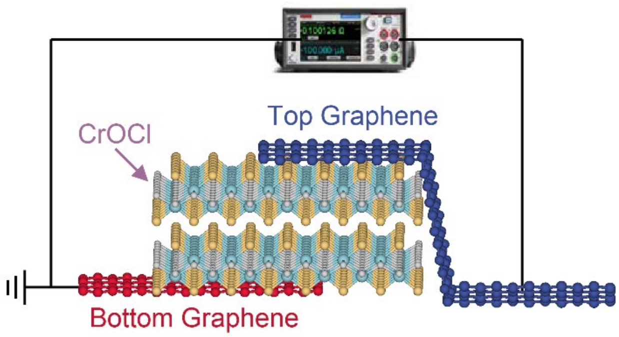

Figure S8. The Measurement method schematic of temperature-dependent tunnelling magnetic conductance. The electrical circuit of the measurement of temperature-dependent tunnelling magnetic conductance.

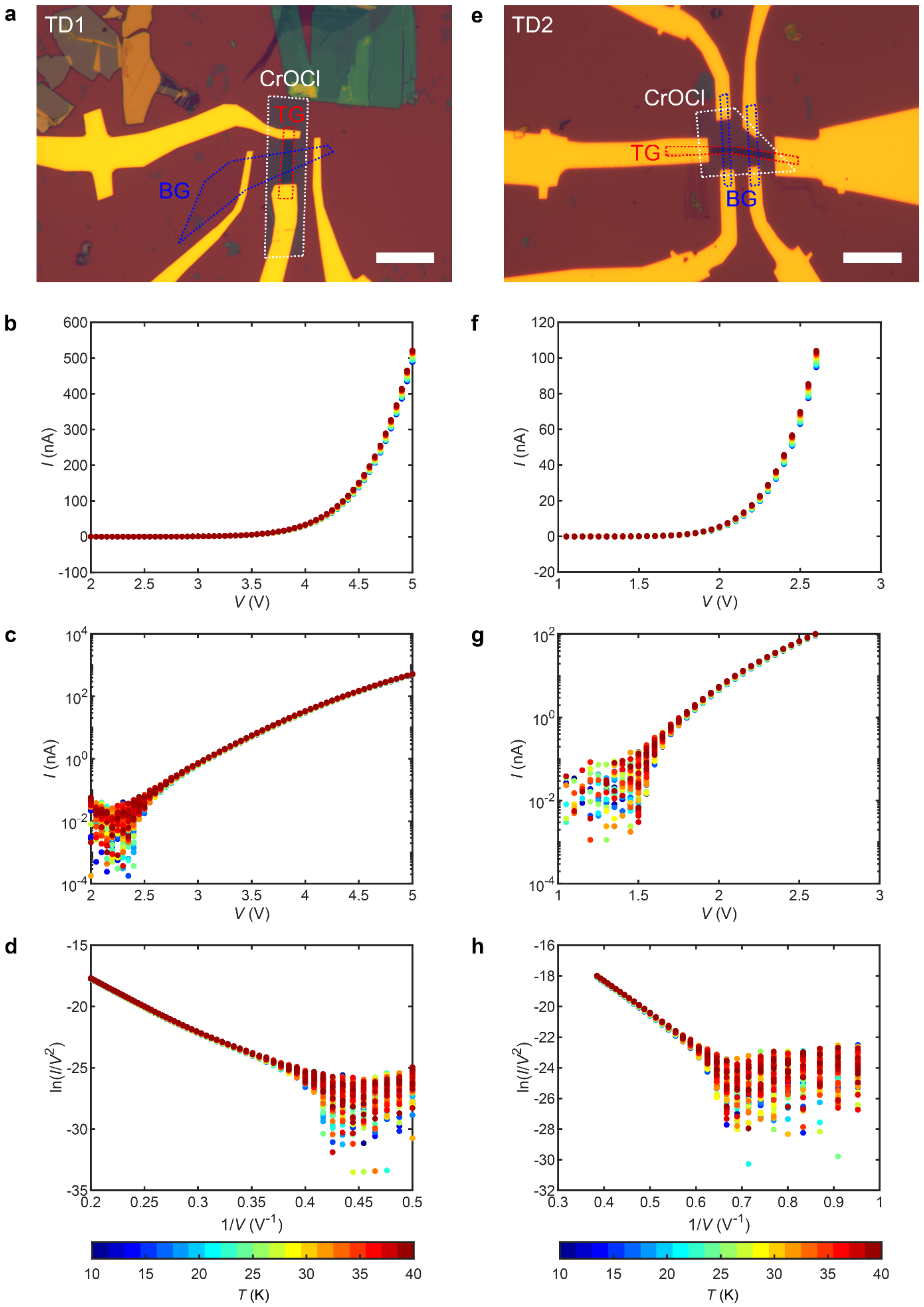

Figure S9. Temperature- dependent tunnelling magnetic conductance of CrOCl. a-h The optical

image (**a**,**e**), I-V curve (**b**,**f**), log(I)-V curve (**c**,**g**) and ln(I/V^2)-V^{-1} curve (**d**,**h**) of the tunnelling magnetic conductance device TD1 (**a**-**d**) and TD2 (**e**-**f**) under different temperatures. The scale bar is 20 μm.

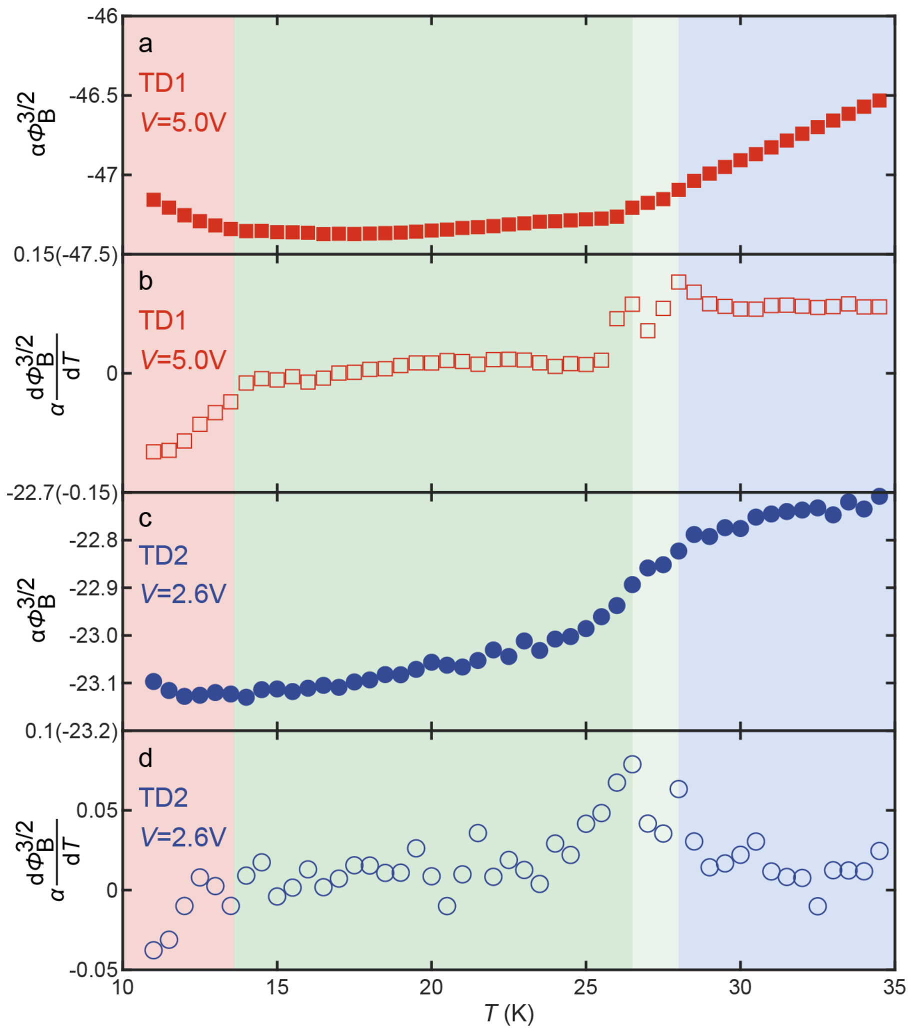

Figure S10. Phase transition associated tunneling magnetic conductance signal. a,c The extracted $\alpha\phi_B^{3/2}$ by fitting the *I*-*V* curves in TD1 (**a**) and TD2 (**c**) at different temperatures. **b,d** The derivative of the extracted $\alpha\phi_B^{3/2}$ with respect to temperature in TD1 (**b**) and TD2 (**d**) at different temperatures.

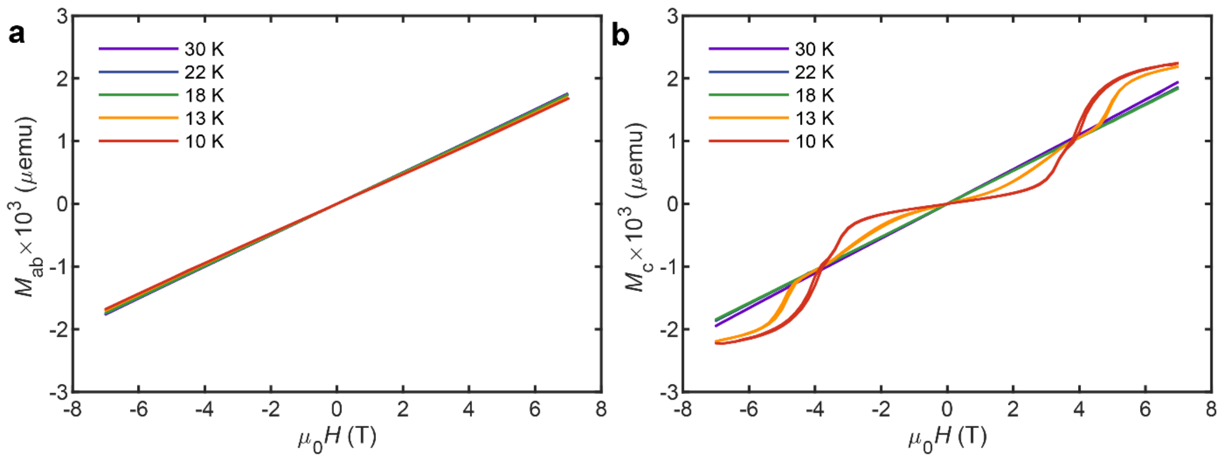

Figure S11. The magnetic field dependence magnetic moment of bulk CrOCl. a,b *M*−*H* curves with the applied field parallel (**a**) or perpendicular (**b**) to the ***c***-axis at different temperatures.

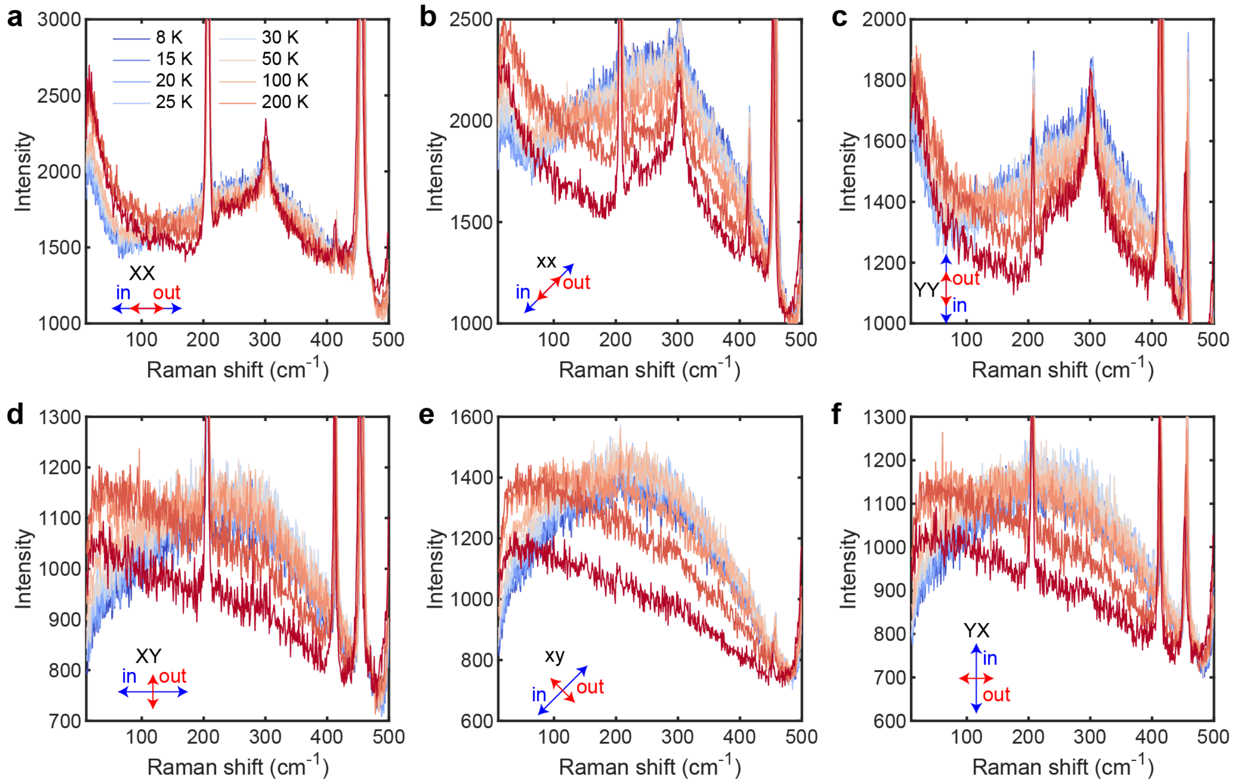

Figure S12. Temperature-dependent Raman spectroscopy of bulk CrOCl in different polarized configurations. a-c Temperature-dependent Raman spectroscopy of bulk CrOCl in different parallel polarized configurations. **d-f** Temperature-dependent Raman spectroscopy of bulk CrOCl in different perpendicular polarized configurations.

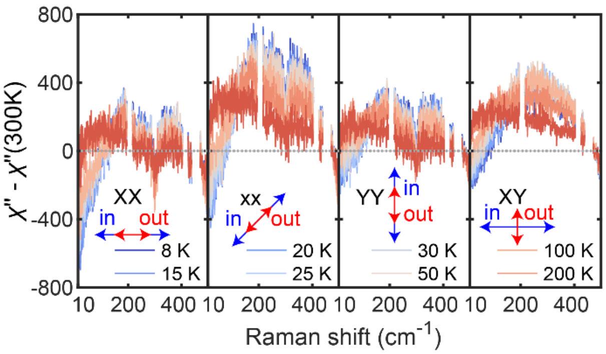

Figure S13. Subtraction in Raman responses between various temperatures and 300K under different polarized configurations. from Left to Right Subtraction in Raman responses between various temperatures and 300K under XX, xx, YY and XY configurations.

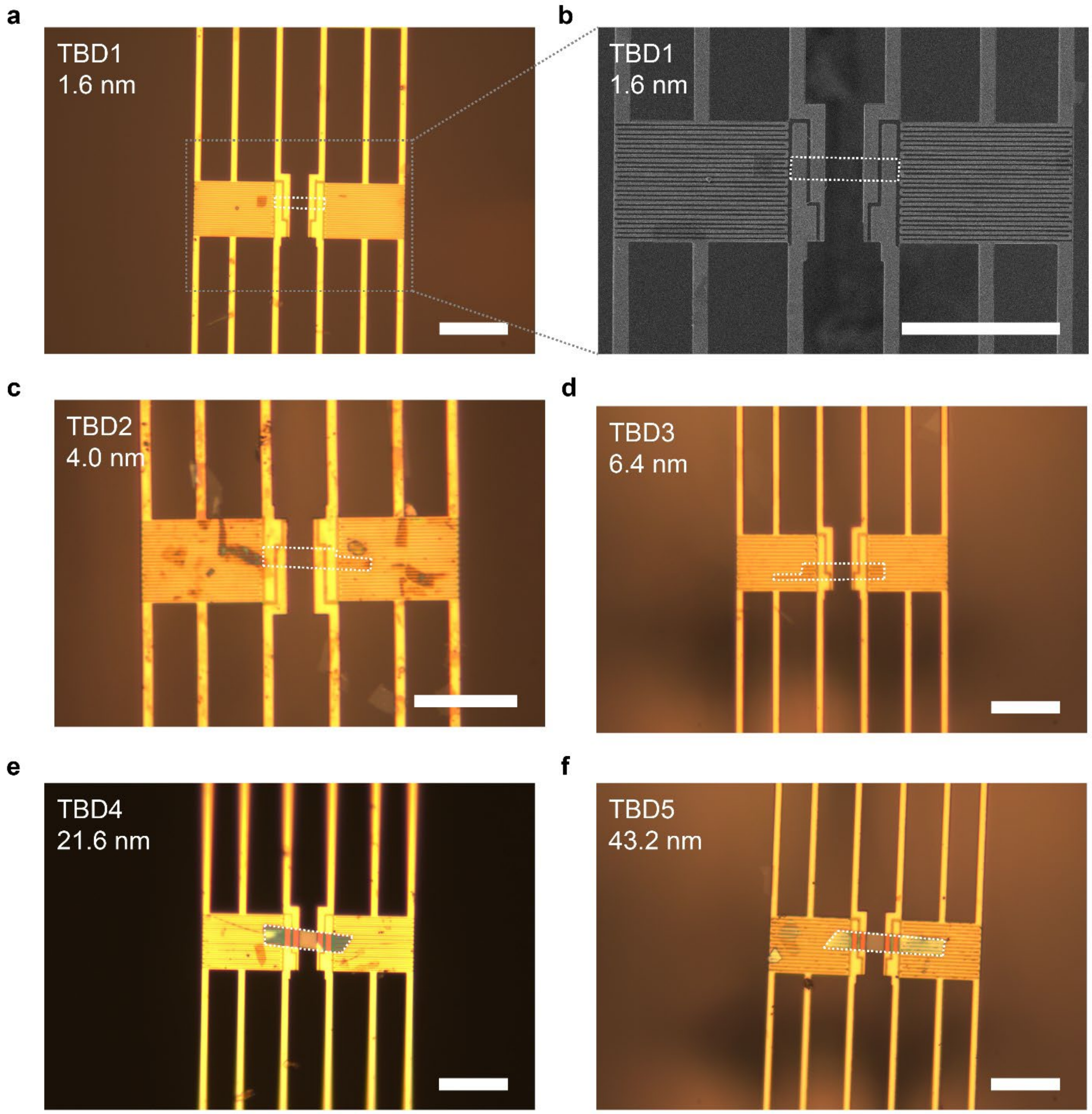

Figure S14. Suspended thermal bridge devices for measuring thermal conductivity with different thickness CrOCl flakes. a,b Optical (**a**) and SEM (**b**) images of a suspended thermal bridge device for measuring the thermal conductivity of a bilayer CrOCl. **c-f** Optical images of the suspended thermal bridge device for measuring the thermal conductivity of FL CrOCl. The scale bar is 20 μm.

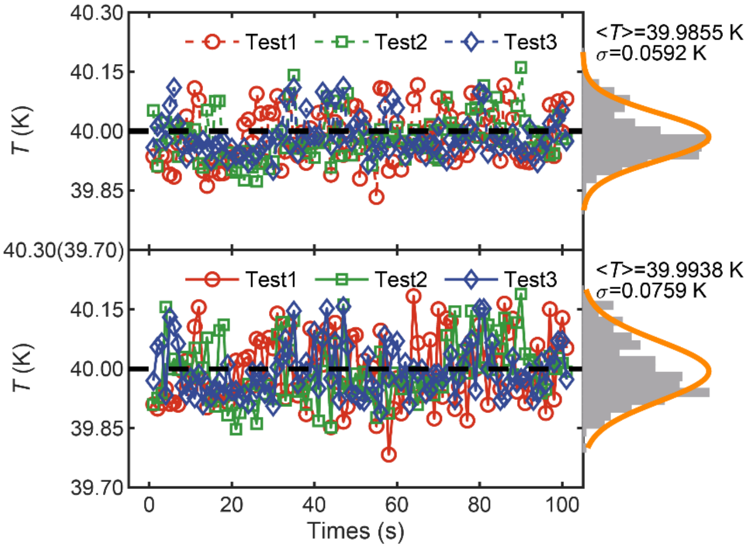

Figure S15. Temperature stability test of the double Wheatstone bridge enhanced suspended thermal bridge method.

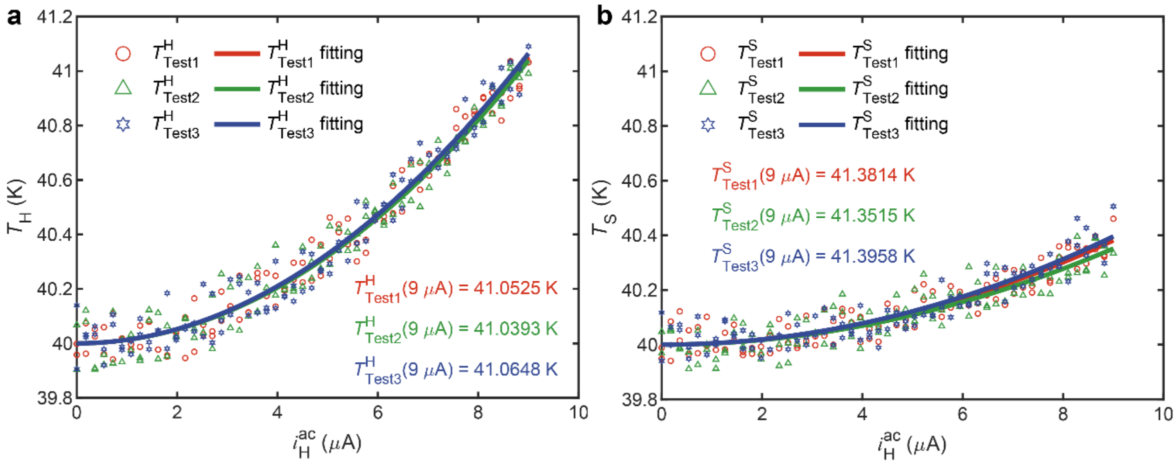

Figure S16. Measurement repeatability test of the double Wheatstone bridge enhanced suspended thermal bridge method. a-b The repeatability of a double Wheatstone bridge enhanced suspended thermal bridge method for the heater (**a**) and sensor membranes (**b**).

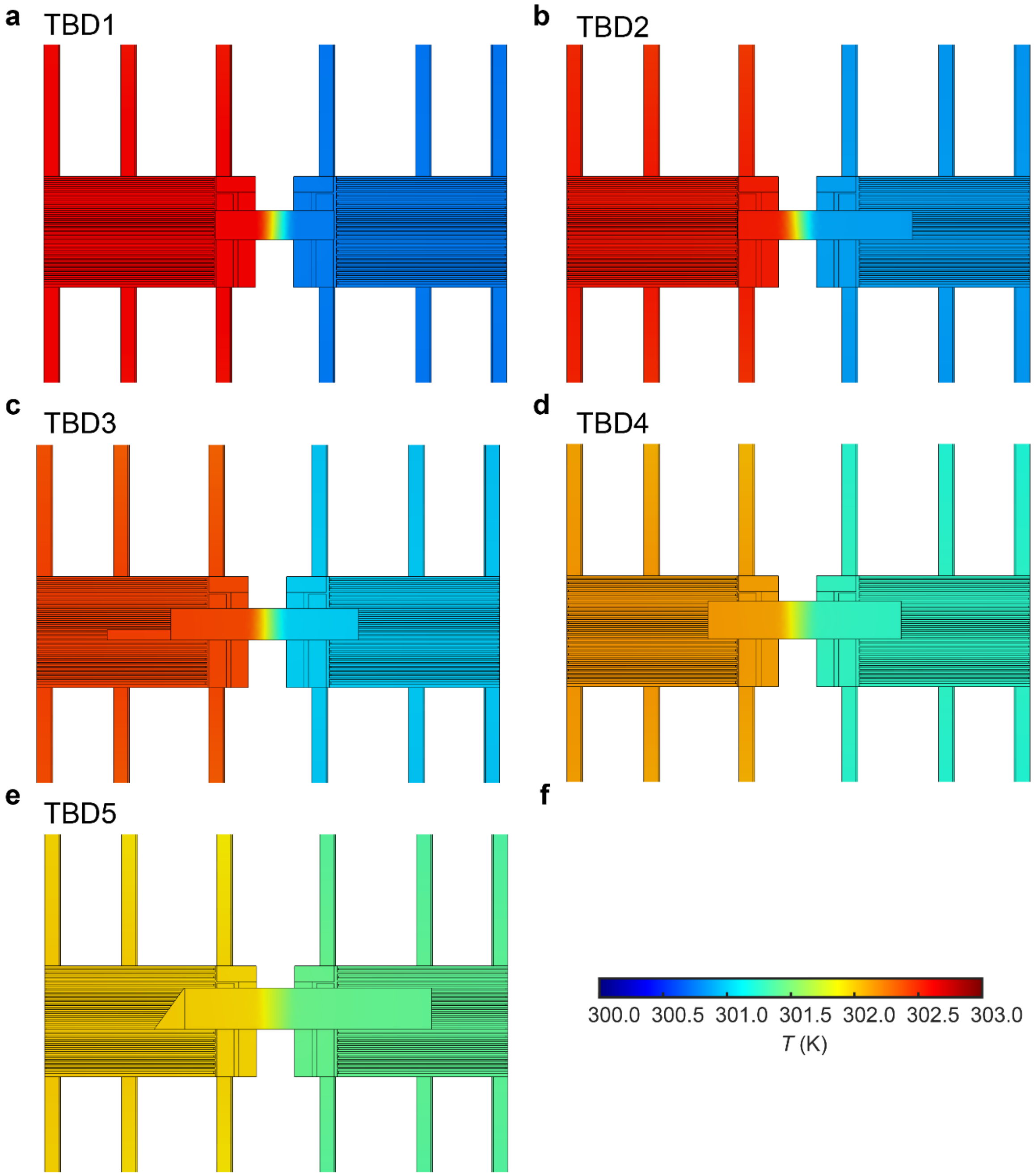

Figure S17. Finite element simulation of double Wheatstone bridge enhanced suspended thermal bridge method. a-e Temperature mapping of double Wheatstone bridge enhanced suspended thermal bridge method obtained by finite element simulation.

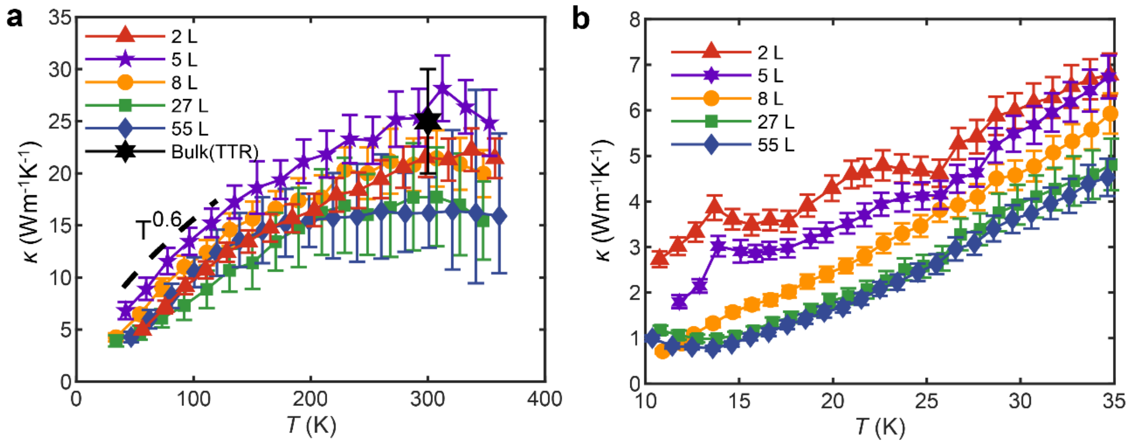

Figure S18. The thermal conductivity of CrOCl with different thicknesses. a The thermal conductivity of different thickness CrOCl flakes from 340 K to 40 K with a step of 20 K. The black hexagonal star indicates the thermal conductivity of the bulk CrOCl measured by TTR method. **b** The thermal conductivity of different thickness CrOCl flakes from 35 K to 11 K with a step of 1 K.

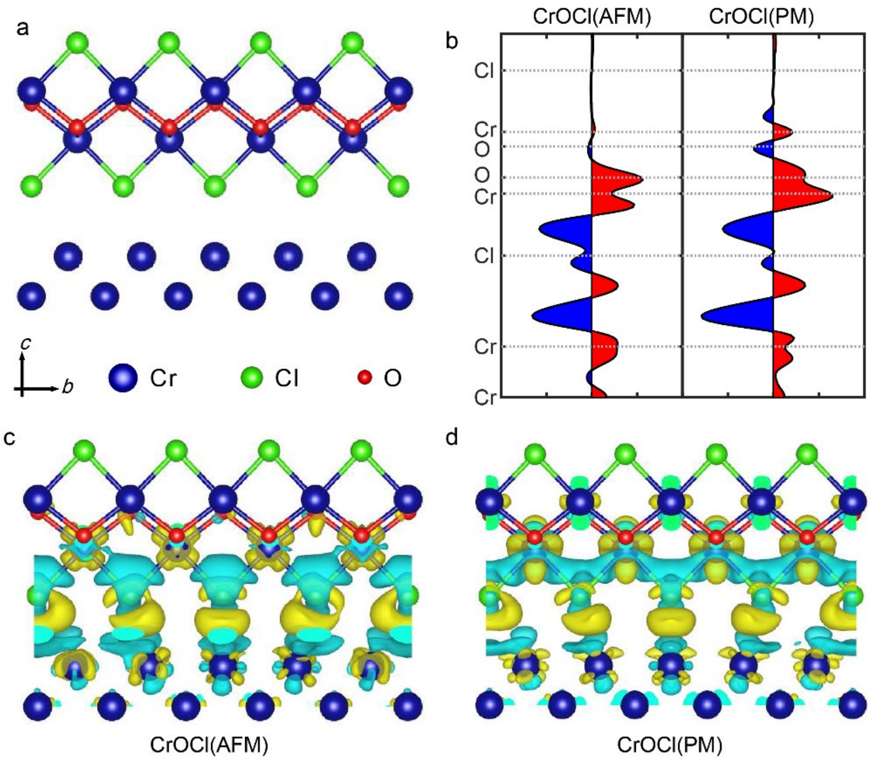

Figure S19. Differential charge density distribution in CrOCl/Cr proximity system. a Side view of CrOCl/Cr proximity system. **b** Differential charge density distribution under CrOCl(AFM)/Cr and CrOCl(PM)/Cr state along the ***c***-axis. **c-d** 3D diagram of the differential charge density distribution under CrOCl(AFM)/Cr and CrOCl(PM)/Cr state.

Methods

Method S1 Untra-sensitive suspended thermal bridge enhanced by double Wheatstone bridge

Since the temperature difference between the two phase transition points is minimal, and the intrinsic in-plane thermal conductivity of CrOCl is very low atthe low temperatures, accurately measuring the thermal conductivity near the phase transition temperature requires meeting two key criteria: 1. Maintaining a small temperature bias to prevent the phase transition induced by the heater heating; 2. Using a highly sensitive thermometer capable of detecting small temperature changes. In the previous work, a voltage-type Wheatstone bridge is introduced at the sensor platform to measure the nanowire with a low thermal conduction, using high current and high temperature bias on the heating platform.[1-5] Although this method improves the measurement precision by increasing the temperature of the heating platform, thereby enhancing the temperature and sensitivity of the sensor platform, it is unsuitable for phase transition system. This is because the temperature of the heating platform often increases by several tens of Kelvin, causing the sample to undergo a phase transition even if the base temperature has not yet reached the phase transition point.

Here, we employa current-type Wheatstone bridge to achieve high temperature sensitivity on both the heating platform and the sensor platform, as illustrated in Figure S15. A large DC current and a small AC current are simultaneously applied to the Wheatstone bridge on the heating platform. The DC current (~uA) creates the temperature bias, while the AC current (~500 nA) enables the temperature measurement. The resistance of the heating (or sensor) platform can be expressed as:

$$R_{\mathrm{H(s)}} = \frac{(i_{\mathrm{H}}^{\omega} R_{\mathrm{H(s)},2} - v_{\mathrm{H}}^{\omega})(R_{\mathrm{H(s)},1} + R_{\mathrm{H(s)},3})}{i_{\mathrm{H}}^{\omega} R_{\mathrm{H(s)},3} + v_{\mathrm{H}}^{\omega}} - R_{\mathrm{H(s)},2}. \tag{S1}$$

Subsequent data processing and error analysis follow the standard procedures used in the normal suspended thermal bridge method. A temperature stability and temperature sensitivity test is shown in Figure S15b, where the temperature fluctuations of the heating platform and the sensor platform are 60 mK and 76 mK, respectively. And ata base temperature of 40K, repeated measurementsyield nearly identical results. In the actual sample measurement, we limited the temperature bias on the heating platform to less than 0.5 K at each base temperature.

Notes

Note S1 The in-plane thermal conductivity of CrOCl at high temperature

In this work, we also measure the in-plane thermal conductivity of CrOCl at high temperature, and the results are shown in Supplementary Fig. 18a. We observe a significantly lower thermal conductivity than that predicted by theory[6] and measured by Raman[7]. The thermal conductivity of CrOCl is approximately 20 $Wm^{-1}K^{-1}$ at room temperature, showing minimal dependency on its thickness.

To further examine the thickness-dependent characteristics of CrOCl's in-plane thermal conductivity, the transient thermoreflectance (TTR) method is employed. This technique involves the deposition of a 20 nm Cr adhesion layer, followed by a 100 nm Au transducer layer on CrOCl. For the TTR measurements, an 8 ns pulse UV laser (355 nm) serves as the heating pump, while a continuous-wave laser (532 nm) is used as the probe, with beam diameters of approximately 70 μm and 2 μm, respectively, at the sample surface. Detailed methodology and setup information can be found in references. [8-10] According to TTR results (also shown in Supplementary Fig. 18a), the thermal conductivity of bulk CrOCl is around 25 W/m·K at room temperature, indicating no significant thickness dependence within the error margin.

Reference

1 Dong, L. *et al.* Dimensional crossover of heat conduction in amorphous polyimide nanofibers. *National Science Review* **5**, 500-506 (2018).

2 Liu, D., Xie, R., Yang, N., Li, B. & Thong, J. T. L. Profiling nanowire thermal resistance with a spatial resolution of nanometers. *Nano Letters* **14**, 806-812 (2014).

3 Wingert, M. C., Chen, Z. C. Y., Kwon, S., Xiang, J. & Chen, R. Ultra-sensitive thermal conductance measurement of one-dimensional nanostructures enhanced by differential bridge. *Review of Scientific Instruments* **83**, 024901 (2012).

4 Yang, L. *et al.* Suppressed thermal transport in silicon nanoribbons by inhomogeneous strain. *Nature* **629**, 1021-1026 (2024).

5 Zheng, J., Wingert, M. C., Dechaumphai, E. & Chen, R. Sub-picowatt/kelvin resistive thermometry for probing nanoscale thermal transport. *Review of Scientific Instruments* **84**, 114901 (2013).

6 Yu, B.-Y. *et al.* Strain effects on the lattice thermal conductivity of monolayer CrOCl: A first-principles study. *Materials Today Communications* **38**, 107665 (2024).

7 Zheng, X. *et al.* Highly anisotropic thermal conductivity of few-layer CrOCl for efficient heat dissipation in graphene device. *Nano Research* **15**, 9377-9385 (2022).

8 Zhou, Y., Dong, Z.-Y., Hsieh, W.-P., Goncharov, A. F. & Chen, X.-J. Thermal conductivity of materials under pressure. *Nature Reviews Physics* **4**, 319-335 (2022).

9 Zhou, Y. *et al.* Barrier-layer optimization for enhanced GaN-on-Diamond device cooling. *ACS Applied Materials & Interfaces* **9**, 34416-34422 (2017).

10 Meng, X. *et al.* Thermal conductivity enhancement in MoS_2 under extreme strain. *Physical Review Letters* **122**, 155901 (2019).
